# How a geriatrician-led emergency department model works in practice: a realist evaluation

**DOI:** 10.1093/ageing/afag036

**Published:** 2026-02-20

**Authors:** Celene Y L Yap, Lannie Ho, George Braitberg, Marie Gerdtz, Michael Murray, Paul Yates, Kathryn Lee, Lee Yung Wong, Cilla Haywood, Sanka Amadoru

**Affiliations:** The University of Melbourne - Department of Critical Care, Parkville, 3010, Victoria, Australia; National Ageing Research Institute Parkville, 3050, Victoria, Australia; Austin Health - Emergency Department, Heidelberg, 3084, Victoria, Australia; Austin Health - Department of Geriatric Medicine, Heidelberg, 3084, Victoria, Australia; The University of Melbourne - Department of Critical Care, Parkville, 3010, Victoria, Australia; Austin Health - Emergency Department, Heidelberg, 3084, Victoria, Australia; La Trobe University – Nursing, Bundoora, 3086, Victoria, Australia; Austin Health - Department of Geriatric Medicine, Continuing Care Division, Heidelberg,3084, Victoria, Australia; Austin Health - Medical and Cognitive Research Unit, Department of Geriatric Medicine, Continuing Care Division, Heidelberg, 3084, Victoria, Australia; The University of Melbourne - Department of Medicine, Parkville, 3010, Victoria, Australia; Austin Health - Department of Geriatric Medicine, Heidelberg, 3084, Victoria, Australia; Austin Health - Emergency Department, Heidelberg, 3084, Victoria, Australia; The University of Melbourne - Department of Critical Care, Parkville, 3010, Victoria, Australia; Austin Health - Department of Geriatric Medicine, Continuing Care Division, Heidelberg,3084, Victoria, Australia; Austin Health - Department of Geriatric Medicine, Heidelberg, 3084, Victoria, Australia; The University of Melbourne - Department of Medicine, Parkville, 3010, Victoria, Australia; Bendigo Health, Bendigo, 3550, Victoria, Australia

**Keywords:** emergency department, geriatric care, realist evaluation, boundary spanning, care transitions, hospital admission, qualitative research, older people

## Abstract

**Background:**

Embedding geriatric medical expertise in the emergency department (ED) is proposed to improve care for older people, yet the underlying mechanisms and context causing impact to remain poorly understood.

**Objective:**

To develop programme theories explaining how, why, and under what conditions a geriatrician-led innovation model improves care for older people presenting to the ED.

**Methods:**

We conducted a realist evaluation of a geriatrician-led innovation in the ED. Data were generated through non-participant observation, document review, interviews and focus groups with geriatricians, ED clinicians, surgical and general medicine teams. Analysis was abductive and retroductive, iteratively configuring data into context–mechanism–outcome configurations to produce programme theories.

**Results:**

Sixteen interviews and five focus groups were conducted. We developed programme theories suggesting that embedding a geriatrician in the ED triggered timely disposition planning, strengthened care transitions, and fostered informal capability building. These outcomes arose through mechanisms of trust-building and boundary spanning, activated in contexts where specialist authority was recognised, trust with ED teams was established, and staffing capacity was sufficient. Limited after-hours coverage and competing ED priorities constrained these mechanisms, resulting in the deferral of some treatment and disposition decisions to the geriatrician, reducing intended efficiencies of timely and coordinated care.

**Conclusions:**

This study advances understanding of how a geriatrician-led innovation model in an ED improve care delivery for older people by identifying the mechanisms and contextual conditions that shape their impact. The theories developed offer a foundation for ongoing theory building, requiring continued testing and refinement across diverse ED settings.

## Key Points

- Geriatrician input improves complex decision-making in emergency care.
- Legitimacy and collaboration enable integration beyond formal hierarchy.
- The GEDI model impact conditioned by timing, workload, and on-site proximity.
- Sustained impact depends on tailored training and cross-team knowledge sharing.

## Introduction

Older people experience longer emergency department (ED) stays, higher rates of iatrogenic complications, and greater risk of functional decline and unplanned admission than younger cohorts, making the ED a high stakes setting for care optimisation [1]. Embedding geriatricians within ED is a promising approach to improving the quality and safety of care for older people presenting with multimorbidity, polypharmacy, and frailty-related syndromes [2–4]. However, there is still limited understanding of how embedded geriatrician models generate improvements in care, which patients benefit the most, and the circumstances in which these models achieve the greatest impact [2].

Multiple, interacting pathways are likely to underpin observed improvements in care for older people, delivered through geriatrician-led interventions, including earlier recognition of geriatric syndromes; risk-stratified decision-making that balances clinical appropriateness with system constraints; deprescribing and medication reconciliation; facilitation of goal-concordant care and advance care planning; and strengthened linkages with community services that avoid unnecessary admissions [4, 5]. However, evaluations that focus solely on outcomes, such as admission rates or length of stay, reveal little about how or why these changes occur. Without examining the underlying mechanisms and the contextual conditions that shape them, it is impossible to explain why benefits emerge in some EDs or patient populations but not others.

Realist methodology offers a potentially useful lens for examining this problem because it seeks to articulate how outcomes arise from mechanisms that are triggered (or inhibited) in particular contexts (context–mechanism–outcome configuration, CMOC) [6, 7]. Rather than treating embedded geriatrician models as uniform interventions, realist evaluation treats them as complex social programmes whose effects depend on the interaction between specialist expertise, team relationships, patient characteristics, and organisational conditions. By explicating these configurations, realist analysis can inform patient selection, and redesign of ED workflows to concentrate specialist attention where it yields the greatest benefit.

Accordingly, this realist evaluation aimed to develop programme theories explaining how embedded geriatrician models improve care for older people in EDs by: [1] identifying key mechanisms through which geriatrician expertise enhances care quality; [2] delineating contextual factors that enable or constrain those mechanisms; and [3] generating CMOCs to account for variation in outcomes across different circumstances.

## Methods

### Study design and setting

The study combined multiple qualitative sources (non-participant observation, document review, interviews and focus groups with iterative theory refinement using teacher–learner cycles). Reporting is aligned to RAMESES II guidance on realist evaluation reporting standards [8].

The study was conducted at a metropolitan tertiary referral hospital in Victoria, Australia, where ED receives more than 90 000 visits annually. A Geriatric Emergency Department Innovation (GEDI) model had been introduced, embedding consultant geriatricians within routine ED workflow. GEDI geriatricians worked proactively alongside the ED clinicians, including the established Emergency Care Coordination Team (ECCT), which comprises allied health and nursing practitioners.

**Figure 1:**
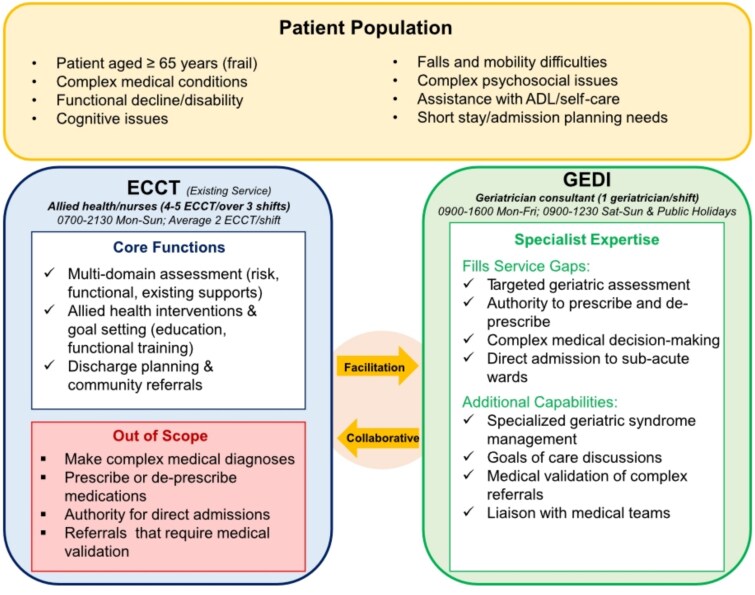
The GEDI model.

The ECCT supports ED patients through multi-domain assessment and coordination of discharge planning, including facilitation of access to domiciliary and community services. The ECCT service is available to all ED patients based on clinical need, rather than being restricted to older people, and operates 7 days a week from 09:00 to 21:00 [9]. In contrast, GEDI geriatricians are rostered exclusively to the ED 7 days a week and provide specialist geriatric medicine input for older patients, with coverage from 09:00 to 16:00 on weekdays and from 09:00 to 12:00 on weekends. Their role includes early senior clinical decision-making, support for safe and timely discharge, facilitation of direct admission to geriatric wards when appropriate, and early linkage with hospital-operated community programmes. A full description of the GEDI model has been published elsewhere [10, 11]. Figure 1 provides an overview of the GEDI model design and its collaborative integration with ECCT.

All GEDI geriatricians are fellows of the Royal Australasian College of Physicians, who have undergone advanced training in geriatric medicine. They are expert in the physiology of ageing and the medical conditions which affect older people. Their training settings include acute hospitals, outpatient clinics, community settings and residential aged care. Their ability to effectively work in the ED draws on this training and knowledge of the services available at the hospital and surrounding health services.

**Figure 2:**
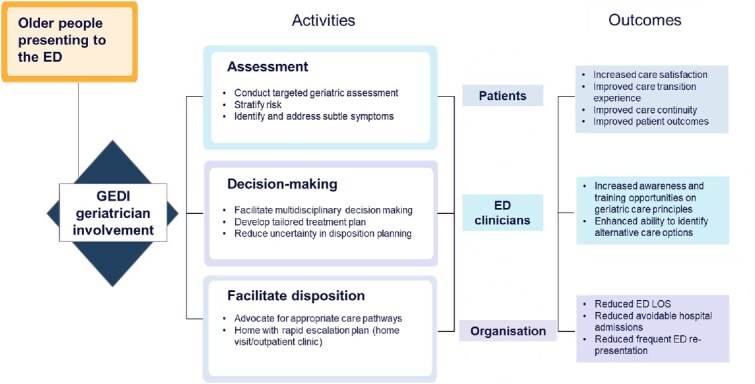
Initial programme theory for the GEDI model presented as a programme-logic diagram.

We began by interviewing the consultant geriatricians who had developed and implemented the GEDI model, to elicit initial programme theories (IPTs) about the resources introduced and their anticipated proximal outcomes (Fig. 2). These preliminary theories were then refined through interviews and focus groups with participants in roles that had interacted with the model, including other geriatricians rostered to the ED as GEDI geriatricians, ED physicians, ED nurses, members of the ECCT, ED pharmacists, inpatient admitting teams, and surgeons. Interviews were conducted using realist teacher–learner cycles, in which context-mechanisms-outcome propositions were presented for participants to confirm, refute, or amend with concrete examples; discrepant cases were actively sought to challenge and deepen emerging explanations [12, 13].

Maximum-variation principles (role, seniority) guided recruitment, with a mix of focus groups (to surface shared logics and within-role variation) and one-to-one interviews (to probe role-specific reasoning). Sampling continued until additional data no longer generated substantively new CMOCs for the focal programme theories [12, 13].

### Data collection

Semi-structured one-to-one interviews and focus groups were carried out from January to March 2025 to accommodate participants’ varying work patterns. All interviews were led by CY, a health service researcher experienced in qualitative methods and not involved in GEDI service delivery. Sessions took place in private hospital rooms or via Microsoft Teams, according to participant preference. Interviews lasted 42–60 min (mean 50 min) and were audio-recorded with consent. Fieldnotes were made during and immediately after each interview to capture contextual details and early analytic impressions. Interview guides were iteratively adapted as theories matured; early interviews prioritised elicitation (‘how/for whom/when’), later interviews targeted refinement of specific CMOCs. All sessions were audio-recorded and professionally transcribed. In addition to interviews and focus groups, we triangulated multiple sources: (i) non-participant observation: CY shadowed a GEDI geriatrician (SA) during his ED shift to document work-as-done, and interactions likely to trigger mechanisms, with contemporaneous fieldnotes; and (ii) document review: the GEDI model handbook and local artefacts (e.g. referral criteria, role descriptions) to identify intended functions, consultation triggers, and inter-service pathways.

### Analysis

Analysis combined abductive reasoning (linking data to plausible explanatory accounts) with retroduction (inferring underlying causal processes) in iterative cycles from IPTs to progressively refined CMOCs. Transcripts and fieldnotes were coded in NVivo software (Lumivero, Version 14) against provisional C, M and O nodes, with open codes for unanticipated patterns. We labelled data fragments by their provisional C/M/O role and collated them into candidate CMOCs. For theory building and refinement, coded data were configured into CMOCs explaining how the GEDI model produced outcomes for patients, ED clinicians, and the organisation under specified conditions. Subsequently, CMOCs across roles were compared to identify demi-regularities and scope conditions. The final set of programme theories was developed iteratively, highlighting how, for whom, and under what conditions the GEDI model contributed to changes in care processes and outcomes. A parallel determinants analysis (barriers/facilitators) using Consolidated Framework for Implementation Research was conducted as a separate workstream and is reported elsewhere [10]. Credibility was enhanced through method triangulation (observation, documents, interviews/focus groups) and role triangulation in sampling.

### Ethics

The study received approval from Austin Health Human Research Ethics Committee (HREC/109608/Austin-2024). Observations were overt; no patient-identifiable information was recorded in fieldnotes.

## Results

A total of 31 participants were recruited. Participant characteristics are summarised in Table 1.

**Table 1:** Demographic and professional characteristics of participants (n = 31).

**Characteristics**	**n (%)**
**Age group (years)**	
25–34 years	8 (26)
35–44 years	11 (36)
45–54 years	10 (32)
55+ years	2 (6)
**Gender**	
Female	25 (81)
Male	6 (19)
**Professional role**	
Emergency physician	5 (16)
Emergency assistant nurse unit manager	4 (13)
Geriatrician	5 (16)
Pharmacist	5 (16)
Admitting registrar/consultant	4 (13)
Emergency care coordination team	8 (26)
**Experience in current role**	
Less than 1 year	5 (16)
1–2 years	4 (13)
3–5 years	8 (26)
6–10 years	7 (22.5)
More than 10 years	7 (22.5)
**Geriatric medicine training**	
Received specific training	8 (26)
**Frequency of interaction with GEDI geriatrician**	
Daily	11 (36)
Occasionally	6 (19)
Weekly	14 (45)
**Self-reported understanding of GEDI’s role**	
Excellent	12 (39)
Good	17 (55)
Moderate	1 (3)
Limited	1 (3)

Two programme theories were developed, each articulated through supporting CMOCs that explain when, how, and for whom the GEDI model influences ED care for older people (Appendix 1). Table 2 summarises the key contexts and mechanisms, and the resultant outcomes, including instances of unintended outcomes.

**Table 2:** Summary context–mechanism–outcome (CMO) configurations that explained outcomes.^*^

**Outcome Domain**	**Outcome**	**Contexts**	**Core mechanisms** ^**‡**^
**Service integration & relational dynamics** ***(implementation outcomes)***	GEDI acceptance & successful integration	ED clinicians work in high-pressure environments rely on trusted geriatric specialists to support complex decision-making (CMOC 1.1a) Dedicated geriatrician is embedded in the ED (CMOC 1.1b) Daily variability in patient mix; need for flexible consultation pathways (CMOC 1.2a)	Perceived clinical credibility and interpersonal trust in GEDI geriatricians legitimise their authority over treatment planning and disposition decisions Demonstrated responsiveness to shifting ED priorities signals that GEDI is ‘working with’ rather than ‘alongside’ the ED, reinforcing relational cohesion
	Relational tensions (−)	When geriatrician plans appear mis-aligned with ED performance targets (e.g. time-to-disposition, length-of-stay) and are seen as unlikely to change immediate treatment or risk (CMOC 1.1c) When there is limited shared mental models of goals between ED clinicians and geriatricians (CMOC 1.1c)	ED physicians resist implementing plans that are perceived as high time–cost within ED throughput targets
**Efficiency & patient flow *(organisation outcomes)***	Reduced unnecessary hospital admissions	ED clinicians with limited knowledge of community options feel discharge is risky for patients require complex discharge planning (CMOC 2.1a) Experienced geriatricians with experience working across multiple sectors (CMOC 2.2b) Community programmes accept referrals when documentation is specialist-led (CMOC 2.2a) Exposure to geriatric risk-stratification reframes safe discharge is feasible (CMOC 2.3a)	Real‐time geriatric endorsement reassures ED physicians that discharge is clinically defensible Geriatricians are confident in navigating complex referral pathways Specialist-initiated referrals are judged credible by receiving agencies, driving more successful referrals to community care Re-calibration of ED clinicians’ internal risk thresholds facilitates supported discharge decisions
	Reduced ED length of stay	Proactive geriatrician autonomy to ‘pull’ complex cases early (CMOC 2.1b) Geriatricians broker direct access to acute and sub-acute geriatric beds (CMOC 2.1d) Consultant-level endorsement of inpatient referrals accelerates decisions of admission (CMOC 2.1e)	Early consultant input compresses diagnostic/disposition timelines Knowledge of bed state plus admission authority circumvents bottlenecks Inpatient teams infer legitimacy when referral carries geriatrician ‘signature,’ expediting transfer
	Increased ED length of stay/reinforced admission default (−)	Single geriatrician triages under surge conditions, complex cases deferred (CMOC 2.1c) After-hours lack of GEDI cover (CMOC 2.2c)	Geriatricians prioritise ‘quick wins,’ leaving patients require comprehensive assessment to wait ED clinicians fall back on risk-averse admission when no referral channel is available
**Quality of care & continuity *(patient outcomes)***	Timely medication optimisation	ED doctors reluctant to alter medication regimens; view medication optimisation as primary care providers role (CMOC 2.2d)	Geriatricians have expertise in polypharmacy plus cross-sector experience legitimises on-the-spot optimisation, reduce delay or missed opportunities in medication optimisation
	More coordinated & proactive inpatient care	Older adults with multiple comorbidities often have unpredictable trajectories following acute illness (CMOC 2.2e)	Experienced geriatricians can mentally simulate likely clinical trajectories & initiate early goals-of-care discussions, aligning ward care with patient needs from ED onwards
**ED clinicians capability & skill development *(ED clinicians outcomes)***	Strengthened discharge-planning capability	Senior ED physicians witness safe geriatrician-led discharges to community services (CMOC 2.3b)	Indirect learning shifts cognitive schema: discharge becomes a responsible, knowledge-based option, ED clinicians are motivated to actively seek pathway information
	Upskilling of ED clinicians (incl. ECCT)	ECCT previously operates without dedicated geriatrician support (CMOC 2.3c)	Side-by-side work with geriatricians provides authoritative validation and ‘live’ teaching, broadening syndrome-management repertoire and job satisfaction
	Over-reliance on specialist service & potential deskilling (−)	Under time pressure, senior ED physicians perceive geriatric issues as outside emergency remit (CMOC 2.3d)	Responsibility is cognitively outsourced to GEDI; ED physicians defer rather than engage, leading to potential erosion of their own geriatric competence
	Care deferral to SSU/general wards for next-day review (−)	Junior night-shift ED physicians lack confidence and limited access to consultants; no geriatrician cover (CMOC 2.3e)	Uncertainty and perceived risk triggers defensive admission for ‘morning review,’ perpetuating admission volumes and limiting experiential learning

*Abbreviations: ED = emergency department; GEDI = Geriatric Emergency Department Innovation; ECCT = Emergency Care Coordinator Team; SSU = Short-Stay Unit; CMOC = Context–Mechanism–Outcome configuration.*

*(−) negative unintended outcomes*

* A full CMOC matrix is provided in Appendix 1; ‡ Mechanisms are expressed as the underlying generative processes triggered by those contexts.

### Programme theory 1: trust building through clinical and relational credibility and service adaptability

Across roles, participants described how geriatricians built trust with ED clinicians by demonstrating clinical credibility in assessing older people with complex ED presentations and providing clear, contextually relevant treatment plans. This credibility was reinforced through consistent responsiveness to ED priorities.

*“GEDI would be like, “Look, we’ll get them referred to this programme tomorrow, but for now, we’d be happy for them to go to short stay.” Whereas we might have been thinking not for short stay. We’ll never get them home. GEDI would sort of give us the confidence to go, look, we’ve got an exit strategy from short stay tomorrow. I think that GEDI and ED would be more aligned in those respects. . .”* (ED Registrar 2).

*‘...having a geriatrician allows the flexibility because there are a lot of different things that the geriatrician can do. The difficulty with predicting how much need for a specific thing in emergency where the needs of the day often change from day to day...’* (Geriatrician 3).

Relational trust developed through approachable communication styles, willingness to explain geriatric care principles, and visible commitment to shared patient outcomes and the geriaction’s onsite accessibility within the ED. Their ready presence and responsiveness reinforced perceptions of support and reliability, encouraging ED clinicians to actively engage with geriatricians’ input and facilitating integration of the role into routine decision-making.

*“ They’re really easy to talk to. So often I’ll say, ‘Hey, what’s happening? Why are we doing this here?’ And they’ll explain...”* (ED Consultant 2).

While timely, goal-aligned input strengthened trust and collaboration, participants noted that when geriatrician assessments were perceived as overly time-consuming or misaligned with ED operational priorities, it could erode confidence in the GEDI geriatrician’s role.

*‘If they were going to do an assessment that took an hour or over an hour, it would actually make us think a bit more about whether GEDI actually is needed, because then it means the person’s there a bit longer.…’* (ED Consultant 2).

### Programme theory 2: boundary spanning

#### Timely disposition planning & improved care transition

Experienced geriatricians draw on cross-sector clinical experience and established relationships with acute wards, subacute units, and community services. While this cross-sector knowledge reflects core geriatric medicine training and experience, the GEDI role enabled these capabilities to be applied within ED workflows. This boundary-spanning function supported more timely and appropriate disposition planning by facilitating direct connection to suitable care destinations without unnecessary delay.

*‘…the understanding of what’s available in the community, what that looks like in real practice is really helpful in being able to be confident and discussing and explaining the risks of discharges. And also working on the subacute wards and making some of those complex discharge decisions…is something that does come with a lot of experience…’* (Geriatrician 3).

This capability also improved care transitions, with geriatricians ensuring that critical information was shared with receiving teams.

*“…I like how they liaise with the ward team; they do communicate with Ward 10 (acute geriatric medicine ward) very quickly…they hand over, they’re very efficient in facilitating the patient transfer…”* (ED Nurse Unit Manager FG1).

Participants described how geriatricians’ established relationships with downstream services enabled access to pathways and follow-up arrangements that ED clinicians could not initiate on their own.

*‘…Older Person’s Complex Care, which whilst we can refer to them, the GEDI would go oh yes, I’ll get XXX to see them on Tuesday. And that becomes the plan which I would not have the authority to be able to say that….’* (ECCT FG1).

In addition, ED clinicians emphasised that geriatricians’ recognised specialist expertise and consultant physician status conferred professional authority that accelerated referrals and secured timely action, reducing delays in care transitions.

“*…the GEDI team, because it’s consultant-run and because they’re specialists, they’ll have the respect and the ability to make referrals, and those things happen much more easily and quickly than we do…*” (ED Consultant 1).

*‘We really want the geriatrician there for the, “We don”t know if this patient needs to be admitted or not’ kind of person*.’ (ED Consultant 2).

### Informal capability building

In parallel, their visible, collaborative approach fostered informal capability building. ED physicians described becoming more aware of community care options following the introduction of GEDI model. However, this increased awareness sometimes led to reduced confidence or greater nuance in their own disposition decisions.

*‘There are some patients that I would have said even now, or definitely before GEDI, I would have said this patient definitely needs admission. No two ways about it, but I’m sort of changing that a bit now, after landing on someone else and being like, oh no, I think we can send them home.’* (ED Consultant 1).

Some ED physicians became more open to learning about referral options.

*‘...I would like to see some overview slides of some of the key programmes, just so that when I see patients, I have a little bit more of an idea that I could then refer them to. I think it would be interesting to know a little bit more about what the community can offer.’* (ED Registrar 2).

In these instances, the presence of the geriatrician acted as both a resource (modelling new ways of working) and a trigger for changed reasoning (increased caution or curiosity) but did not always lead to behaviour change unless supported by accessible referral pathways or practical knowledge.

*‘I can refer, but is it going to be three days that they get seen or three weeks or three months? I don’t know. What’s the follow-up and how does that work? I don’t know.’* (ED Consultant 2).

Importantly, this proximity also enabled reciprocal learning, as geriatricians were concurrently upskilled through exposure to ED workflows, priorities, and communication styles, enhancing mutual understanding and reinforcing collaboration over time.

*‘Different from being a community geriatrician or an inpatient geriatrician. I think [working as a GEDI geriatrician] helped us develop more of a mutual respect for what our colleagues [in the ED] do and the pressures they have. And I think often, because we can chat, we do share those stresses as well.’* (GEDI Geriatrician 4).

## Discussion

Building on our prior qualitative analysis of GEDI implementation determinants [10], this study extends the analysis by using realist philosophy of science to explain how, why and for whom the model works in practice. The programme theories developed here elucidate where geriatrician input yields the greatest marginal benefit and clarify the mechanisms through which GEDI contributes to observed efficiencies in emergency care, such as reduced hospital admissions and shorter ED stays. Mechanisms such as clinical legitimacy and system navigation through which geriatricians influence ED physicians’ confidence and decision making appear particularly salient in cases involving complex discharge needs and diagnostic uncertainty. These CMOCs support more targeted and strategic allocation of geriatricians’ involvement to patients most likely to benefit. The findings also highlight opportunities to enhance model efficiency, e.g. by delegating initial referral screening to ED clinicians using structured frailty assessments [14–17], thereby preserving geriatricians’ time for high-level clinical decision-making. Such adaptations may improve the model’s effectiveness and sustainability but will require local tailoring and ongoing training to support ED clinicians in incorporating frailty assessment into early clinical decision-making and identifying the subset of older people most likely to benefit from geriatrician input.

Geriatricians frequently facilitated direct discharge from the ED, and findings from our interrupted time series study [11] suggest this may have contributed to reductions in hospital admissions and shorter ED stays. However, these patterns did not appear to extend to short-stay unit (SSU) admissions, which remained stable across the study period [11]. The CMOCs explain how the GEDI model may generate outcomes such as timely disposition planning and improved care transitions, which would be expected to reduce SSU use. However, this anticipated reduction was not observed in the quantitative analysis, suggesting that countervailing mechanisms may have been operating. Limited staffing capacity, only one geriatrician available during business hours likely constrained the model’s reach within a 24/7 ED service. Participants described how decisions made later in the day or under time pressure often limited opportunities for safe discharge, tipping the balance toward SSU admission even when earlier review by the GEDI geriatrician might have supported discharge home. These offsetting mechanisms, one promoting efficiency through proactive planning, the other favouring short-stay admission under constrained conditions may have resulted in a net neutral quantitative outcome. Future evaluation could empirically test this hypothesis through comparing SSU utilisation patterns across EDs or time periods with differing levels of GEDI geriatrician coverage (e.g. weekday vs after-hours service), to examine whether observed variations align with the theorised CMOCs.

A prominent benefit of the GEDI model for patients without acute medical issues was its ability to shift the traditional role of the ED as a ‘catch-all’ safety net toward facilitating timely linkage to appropriate community-based supports to enable appropriate discharges. This function, however, depends heavily on the geriatrician’s specialist, real-time knowledge of care-coordination pathways and service navigation. Assessing the suitability and eligibility of inpatient and community programmes requires awareness of rapidly changing service capacities and referral criteria, information that fluctuates from hour to hour and month to month. Consequently, it is not reasonable to expect ED clinicians to emulate this component of the geriatrician role, as such expertise is built through years of experience across diverse geriatric and community service contexts. These contextual realities highlight why the GEDI model’s effectiveness relies on geriatricians’ system-wide situational awareness rather than transferable procedural skills alone. Hence, indicators such as the number of avoided admissions for non-medical reasons, the proportion of referrals accepted by community services, time to community service access, uptake of recommended supports, and re-presentation rates stratified by disposition pathway would offer a more accurate picture of the effectiveness of the geriatric-specific emergency care models. These alternative metrics might better reflect the realities of geriatric emergency care than length of stay and illuminate outcomes including enhanced complex care coordination, cost avoidance through safe community-based management, and improved quality of care transitions.

Trust-building emerged as more than a relational benefit; it functioned as a mechanism that enabled geriatricians to embed their input within ED workflows and influence clinical decision-making, capability building and service delivery. This foundation of trust created the conditions for effective interdisciplinary collaboration and opened the way for a second, complementary programme theory: boundary-spanning. In this role, geriatricians were able to mobilise resources, align care priorities, and coordinate transitions across professional and organisational boundaries, thereby enhancing integration across the continuum of care. Our findings align with boundary spanning theory, which emphasises that an individual’s position within the organisational hierarchy strongly shapes their capability to bridge professional, organisational, and sectoral divides [18–20].

Previous studies, including a review of boundary spanning roles in organisations [20], suggest that higher-status positions confer formal authority and recognised expertise, enhancing credibility, access to resources, and acceptance by colleagues. In geriatrician consultant–led or multidisciplinary models, the presence of at least one team member with specialist status appears to reduce resistance to integration within the ED, as this positional authority can legitimise recommendations, expedite negotiations with ED staff and other specialties, and facilitate coordination across service boundaries [16, 21]. Consistent with this, participants in our study described how the geriatrician’s recognised expertise and professional authority supported cross-boundary collaboration and legitimised new pathways of care. However, these effects appeared to operate less through hierarchical status alone and more through how expertise, communication style, and consultative leadership interacted with entrenched referral practices, team culture, and workflow pressures. In this way, legitimacy operated as a relational mechanism, whose activation depended on contextual factors such as team culture, leadership style, and existing referral practices.

Physical proximity emerged as an important contextual enabler of the mechanisms underpinning the GEDI model integration. Having the geriatrician located within the ED facilitated informal communication, rapid consultation, and timely joint decision-making, which ED staff described as fostering a sense of shared team membership and mutual trust. This co-location allowed the geriatrician to observe patient flow, understand real-time operational pressures, and adapt their input to the ED’s dynamic environment. Participants contrasted this immediacy with models relying on remote consultation or telephone advice, noting that accessibility and visibility were integral to sustaining engagement and perceived value of the role. These findings suggest that proximity acts as a contextual condition that enables mechanisms of responsiveness, collaboration, and legitimacy to operate effectively within the ED.

By contrast, models led by other healthcare professionals, such as nurse-led or allied health–led services may still activate boundary spanning mechanisms if trust-building has been established early in implementation [18, 22, 23]. However, consistent with empirical work in public health and organisational studies, lower-status roles may have diminished influence in vertical decision-making, face greater challenges in gaining acceptance from higher-status clinicians, and rely more heavily on informal strategies or formalised structures to sustain cross-boundary work [18, 19]. In this context, positional authority should be understood not as an intrinsic property of professional rank, but as a form of legitimacy conferred by others, e.g. when specialist physicians recognise and endorse another clinician’s input or decision-making authority [16, 23]. Thus, how the role is perceived and accepted within existing professional hierarchies, rather than the role title itself, determines the extent to which boundary-spanning mechanisms are activated. These dynamics suggest that while trust-building can partially offset hierarchical disadvantage, endorsement by consultant-level clinicians remains a powerful enabler of cross-boundary effectiveness in acute care settings.

Our findings highlight mechanisms of relational brokerage and ad hoc knowledge exchange that fostered informal capability building, broadening ED clinicians’ perceptions of the feasibility of hospital avoidance and safe discharge for older people. The embedded geriatrician appeared to raise awareness among senior ED clinicians, yet this attitudinal shift did not consistently translate into behaviour change, constrained by perceived gaps in knowledge, time and confidence. Consistent with realist accounts of partial mechanism activation in unsupportive contexts [24], structured supports and role-tailored training are needed to convert awareness into action. In this study, participants proposed a range of strategies through which knowledge exchange between emergency and geriatric medicine teams could be strengthened. For experienced emergency physicians, the priority is system navigation, ease of referral, and referral confidence (awareness of community support programmes, familiarity with referral proformas, and appropriate referrals with ECCT support when geriatric medical input is unavailable). Junior physicians were seen as benefiting from further training in recognising common geriatric syndromes and holistic care principles, including making clear, early referrals to geriatric medicine and avoiding low-value investigations that prolong ED stays. Emergency care coordinators were viewed as key to strengthening discharge planning and community linkage, while ED nurses could be supported through ECCT-led instruction in gait and mobility assessments to inform safe discharge decisions. Formalising these knowledge-sharing strategies could further extend the geriatrician’s boundary-spanning role and promote sustainable practice change.

### Strength and limitations

Our programme theories were developed within one metropolitan, publicly funded ED operating an embedded, in-hours geriatrician model with offsite geriatric medical wards and established community outreach programmes. These conditions may limit transferability to settings with different service models (e.g. consultative or on-call geriatrics), resource envelopes, or policy environments (e.g. rural or private EDs, limited community support programmes). In addition, the evaluation was conducted in a setting where the GEDI model had been sustained in routine practice, despite recognised sustainability challenges described elsewhere [10]; perspectives may therefore reflect experiences of a relatively well-functioning intervention and may differ in contexts where implementation was less successful or not sustained.

While clinician perspectives offered valuable insights into how geriatrician-embedded models are perceived to improve care, relying on this single data source limited our ability to build a complete picture of the programme theories underpinning the model, particularly in relation to why some patients re-present to the ED after discharge. Future work incorporating the perspectives of older patients and their carers will be important to capture how they experience care transitions, what resources and constraints shape their reasoning, and how these factors influence re-presentation patterns. Despite these limitations, this study provides a methodologically rigorous foundation for understanding how embedded geriatrician models improve care for older people in the ED setting. The programme theories offer testable hypotheses for future research and practical insights for clinicians seeking to design targeted interventions for older people presenting to the ED. This work represents an essential first step toward building a more comprehensive evidence base about embedded specialist models and their impact on geriatric emergency care.

## Conclusions

This study contributes programme theories on how geriatrician-led innovations in the ED can shape clinician reasoning and inter-team collaboration to improve care for older people. By explicating the contextual conditions that enable or constrain these mechanisms, our findings advance understanding of how geriatric medical expertise can be integrated into emergency care. These theories provide a foundation for informing the design and adaptation of geriatrician-led models, while ongoing realist inquiry across diverse ED settings will be essential to further refine and extend them.

## Supplementary Material

aa-25-2955-File004_afag036
