# Correction to: How a geriatrician-led emergency department model works in practice: a realist evaluation

**DOI:** 10.1093/ageing/afag194

**Published:** 2026-06-29

This is a correction to: Celene Y L Yap, Lannie Ho, George Braitberg, Marie Gerdtz, Michael Murray, Paul Yates, Kathryn Lee, Lee Yung Wong, Sanka Amadoru, Cilla Haywood, How a geriatrician-led emergency department model works in practice: a realist evaluation, *Age and Ageing*, Volume 55, Issue 2, February 2026, afag036, https://doi.org/10.1093/ageing/afag036

The following changes have been made to the originally published paper.

The order of the last two authors has changed from:

“Celene Y.L. Yap^1,2,3^, Lannie Ho^4^, George Braitberg^1,5^, Marie Gerdtz^6^, Michael Murray^7^, Paul Yates^8,9^, Kathryn Lee^4^, Lee Yung Wong^5^, Sanka Amadoru^4,9,10^, Cilla Haywood1,7” to read: “Celene Y.L. Yap^1,2,3^, Lannie Ho^4^, George Braitberg^1,5^, Marie Gerdtz^6^, Michael Murray^7^, Paul Yates^8,9^, Kathryn Lee^4^, Lee Yung Wong^5^, Cilla Haywood^1,7^, Sanka Amadoru^4,9,10^”

The following statement has been added to the end of the **Acknowledgements** section: “The authors would also like to express their sincere gratitude to Dr Abdi Osman for his invaluable contributions to this research. His expertise and support were instrumental in the development of the study protocol, ethics submission, participant recruitment, and data collection.”

