# Supplementary material for: How a geriatrician-led emergency department model works in practice: a realist evaluation
Source: Age Ageing. 2026 Feb 20;55(2):afag036. doi: 10.1093/ageing/afag036 (PMC13312032; doi:10.1093/ageing/afag036)
Supplement: aa-25-2955-File004_afag036 [file aa-25-2955-file004_afag036.docx]

**How a Geriatrician-Led Emergency Department Model Works in Practice: A Realist Evaluation**

**Appendix 1**. Refined GEDI programme theories expressed as context–mechanism–outcome configurations (CMOCs)

| **CMOC** | **Context** | **Mechanism** | **Immediate Outcome** | **Medium Term Outcome** |
| --- | --- | --- | --- | --- |
| **Programme Theory 1: Trust Building** | | | | |
| **Mechanism 1.1 Clinical and Relational Credibility** | | | | |
| CMOC 1.1a | When ED clinicians work in high-pressure environments characterised by fragmented care pathways and professional hierarchies, they rely on trusted colleagues to guide complex decision-making | GEDI geriatricians demonstrate clinical expertise and build strong interpersonal relationships, ED clinicians perceive them as credible and trustworthy | This perception gives geriatricians the professional legitimacy to influence care decisions and coordinate action across service and disciplinary boundaries | Model acceptance and successful integration |
| CMOC 1.1b | When a dedicated geriatrician is embedded in the ED with protected time and visible support from hospital leadership | The consistent presence of geriatricians during GEDI shifts reduces perceived barriers to consultation and increase willingness of ED clinicians to involve geriatricians in patient care decisions | Input from the GEDI geriatrician is readily sought and incorporated as part of routine ED care for older people |  |
| CMOC 1.1c (-) | When geriatrician lacks familiarity with ED priorities such as time pressures and disposition targets, and request assessments or investigations that could be deferred to outpatient follow-up | ED physicians perceive the geriatrician’s plan to be misaligned with the operational realities of emergency care | ED physicians are reluctant to adopt recommendations from geriatricians | Tensions emerge between GEDI geriatricians and ED clinicians, undermining relational trust |
| **Mechanism 1.2 Service adaptability** | | | | |
| CMOC 1.2a | ED priorities and patient mix vary daily (e.g., surges, staff shortages, variation in clinical presentations). Rigid, protocol-driven services might struggle to integrate because they cannot align with the shifting demands of ED priorities or workflows | When GEDI geriatricians respond flexibly to emergent needs without relying on rigid referral pathways, ED clinicians come to view them as responsive and attuned to ED pressures. This fosters a sense that GEDI is working with the ED rather than alongside or against it, making staff more open to engaging the service | ED clinicians are more likely to initiate referrals, seek input early, and adopt recommendations from the geriatrician into care plans | Model acceptance and successful integration |
| *Trust building enables GEDI to become embedded in ED, a necessary precondition for boundary-spanning mechanisms to be activated* | | | | |
| **Programme Theory 2: Boundary Spanning** | | | | |
| **Mechanism 2.1 Timely Disposition Planning** | | | | |
| CMOC 2.1a | - ED clinicians feel ill-equipped to make disposition decisions for older people with multifaceted needs that require complex discharge planning - ED clinicians have limited knowledge of alternative care options (e.g., community-based transitional programs, geriatric outreach) - Hospital admission is often viewed as the default "safe" option | Access to GEDI geriatricians during key decision-making windows enables ED clinicians to feel reassured that safe discharge is clinically appropriate and supported | Increased rate of supported discharges or referrals to community support programs | Reduce unnecessary hospital admissions |
| CMOC 2.1b | GEDI geriatricians are perceived by ED clinicians as clinically expert and trustworthy because of their specialist status | GEDI geriatricians are granted the autonomy to proactively select patients where they believe specialist input will most improve disposition planning | Clinically complex patients receive timely GEDI input, and discharge planning is initiated earlier in their ED episode | Reduce ED length of stay |
| CMOC 2.1c (-) | When GEDI geriatricians are required to triage referrals under conditions of limited staffing and high service demand, they prioritise cases perceived as most urgent or manageable within available timeframes | High service demand shapes their internal reasoning to focus on rapid impact and risk mitigation, leading to the deferral of more complex cases that require extended assessment and coordination | Older people with multifaceted needs may wait longer or receive less immediate attention | Increase ED length of stay |
| CMOC 2.1d | - No standing pathway allows ED clinicians to book geriatric acute or sub-acute beds directly - General medical ward is the usual default admission destination for older people when GEDI geriatrician is not available | Recognising that GEDI geriatricians have real-time knowledge of subacute bed availability and admission authority, ED clinicians perceive escalation as a reliable and efficient route for timely transfer. This confidence in the process motivates them to refer suitable older people earlier in the episode. | Timely transfer of older people with clear geriatric acute or sub-acute indications to the appropriate ward | Reduce ED length of stay |
| CMOC 2.1e | Within a hierarchically oriented ED, ED clinicians perceive that inpatient teams view ED-generated referrals as clinically “lightweight.” As a result, referrals initiated by ED clinicians alone are frequently questioned or declined, prolonging decision-making and patient length of stay | An embedded GEDI geriatrician (consultant level) co-creates or endorses the referral, contributing recognised specialist expertise and institutional weight. Inpatient admission teams infer that a geriatrician-backed referral is both clinically appropriate and procedurally legitimate, so further verification is unnecessary and accepting it is the lower-risk option | Disposition decisions (admission to acute/sub-acute wards or discharge with services) are made earlier in the ED episode. Patients move to the “right place first,” reducing duplication of assessments | Reduce ED length of stay |
| **Mechanism 2.2 Improve Care Transition** | | | | |
| CMOC 2.2a | Community-based referral services require clear, risk-aligned clinical documentation and often reject referrals that do not meet safety/eligibility thresholds. ED physicians may be unfamiliar with these requirements or under time pressure to meet them | Geriatricians apply advanced clinical assessment skills and are familiar with the expectations, language, and documentation practices of these referral services. Receiving services perceive the referral as credible and appropriately framed. This clarity reduces their uncertainty about risk and eligibility, increasing their confidence in accepting and actioning the referral. | Older people gain timely access to appropriate community support services post ED discharge | Reduce unnecessary hospital admissions |
| CMOC 2.2b | Geriatric care spans multiple sectors (residential aged care, general practice, ED, inpatient wards, community palliative care), each with different priorities, workflows, and referral processes. Experienced geriatricians with experience working across multiple sectors have built deep system-level knowledge, cross-sector relationships, and familiarity with diverse organisational norms | This breadth of experience enables the geriatrician to feel confident in navigating complex referral pathways and making complex discharge decisions | Faster and more effective coordination of care for older people, smoother transitions between care settings, and reduced delays in service access. | Reduce unnecessary hospital admissions |
| CMOC 2.2c (-) | Certain community-based services preferred referrals validated by geriatricians. During after-hours periods, when geriatricians are unavailable, ED clinicians are unable to initiate these referrals. Without access to timely discharge planning support or services, clinicians cannot develop a safe and supported care plan for older people requiring complex transitions | In the absence of viable discharge options, ED clinicians judge that discharging the patient poses unacceptable clinical and legal risks. They perceive hospital admission as the only defensible and safe option. | Default admission to general medical wards or short stay unit for older people, even when clinically unnecessary, due to unresolvable discharge planning barriers during after-hours | Reinforcement of hospitalised as a risk-averse default |
| CMOC 2.2d | ED physicians are often reluctant to modify existing medication regimens for older patients, as they perceive this responsibility lies with primary care providers who have access to longitudinal medical histories and are better positioned to monitor treatment changes over time | Geriatricians contribute specialist expertise in managing polypharmacy and operate across the care continuum, positioning them as trusted clinical decision-makers. Geriatricians perceive medication optimisation, including dose adjustments, substitutions, or cessations as within their legitimate clinical remit. Their confidence in evaluating risk–benefit trade-offs for older people enables them to make timely and appropriate changes to existing regimens during the ED episode | Medication regimens are optimised before ED discharge | Better rationalisation of medication regimens |
| CMOC 2.2e | - Older people with clear indications for acute inpatient admission frequently experience unpredictable clinical trajectories - Acute care clinicians (e.g. ED physicians, trauma surgeons) are trained to manage immediate physiological crises but typically lack the longitudinal/holistic perspective or expertise to anticipate downstream complications - Experienced geriatricians routinely manage longitudinal care needs and are familiar with typical care trajectories in frail older people | Geriatricians draw on tacit knowledge and accumulated clinical expertise to mentally simulate likely clinical trajectories (anticipating complications, rehabilitation needs, and end-of-life considerations), leading to a future-oriented care plan in the ED | Timely initiation of targeted interventions and formal goals-of-care discussions within the ED, rather than waiting for in-ward review | Improved timeliness of clinical decision-making, prognostication, and goals-of-care setting |
| **Mechanism 2.3 Informal Capability Building** | | | | |
| CMOC 2.3a | Emergency medicine physicians generally lack formal training in geriatric care principles, such as recognising and preventing hospital-associated deconditioning, delirium and functional decline, resulting in less tailored assessment and management of older people in the ED | Exposure to geriatric-specific reasoning and informal coaching prompts ED clinicians to reconceptualise older people as requiring tailored risk assessments. This exposure prompts a shift in how they appraise risk and complexity, leading to increased recognition that hospitalisation may not be the safest option for all older people | ED clinicians increasingly avoid risk-averse default hospitalisation in favour of safe discharge | Reduce avoidable hospital admission |
| CMOC 2.3b | Senior ED registrars and consultants, who bear ultimate responsibility for disposition decisions often lack detailed knowledge of alternative community-based transitional programs (e.g. geriatric outreach, transitional care services) | When these experienced ED physicians observe cases in which geriatricians successfully coordinate safe discharges via community services, they reappraise “discharge” as a responsible, supported option. This reappraisal increases their cognitive openness to acquiring knowledge about community services | ED physicians become more motivated to seek information about community-based options, including referral criteria and care pathways, to facilitate safe discharge | Strengthened ED physicians discharge planning capability |
| CMOC 2.3c | The ECCT are historically the main team in the ED with substantive knowledge of geriatric syndromes. As allied-health/nurse-trained professionals they face scope-of-practice limits and lack an on-site medical colleague with geriatric authority for consultation or case verification | Embedded GEDI geriatricians contribute specialist diagnostic insight, system knowledge and professional legitimacy, and work side-by-side with ECCT in developing patient-centred care plans. ECCT recognise this collaboration as authoritative validation and a rare experiential learning opportunity, which reduces clinical uncertainty and fosters confidence in decision-making. | ECCT experience higher job satisfaction, feel professionally empowered, and broaden their repertoire of approaches for managing geriatric syndromes | Upskilling ED clinicians |
| CMOC 2.3d (-) | Under intense time pressure and with limited staffing or diagnostic resources, senior ED registrars and consultants often view the management of older people with complex needs as beyond the remit of “rapid flow” emergency care | Faced with this perceived mismatch between their scope and the patient’s needs, ED physicians cognitively “outsource” responsibility by deferring decision-making to the GEDI geriatrician rather than engaging in shared decision-making or pursuing their own geriatric upskilling | GEDI geriatricians effectively become the default treating clinician for older people in the ED | Over-reliance on the specialist service and potential deskilling of ED staff |
| CMOC 2.3e (-) | ED physicians who face night-shift scenarios where GEDI geriatricians are unavailable, and they lack confidence in making independent decisions | Confronted with uncertainty and the perceived risk of a premature or unsafe discharge without specialist input, these ED physicians default to deferring responsibility rather than decide independently | Patients are admitted to the Short Stay Unit for next-day GEDI review or, in the absence of immediate specialist input, hospitalised as a risk-averse default | Care deferral, maintaining or increasing SSU or general medical wards admission rates |
